# (Re)Conceptualising ‘good’ proxy decision-making for research: the implications for proxy consent decision quality

**DOI:** 10.1186/s12910-022-00809-5

**Published:** 2022-07-18

**Authors:** Victoria Shepherd

**Affiliations:** Centre for Trials Research, Cardiff University, 4th Floor Neuadd Meirionnydd, Heath Park, Cardiff, CF14 0GB UK

**Keywords:** Cognitive impairment, Research, Decision making, Proxy, Third party consent, Decision support

## Abstract

People who are unable to make decisions about participating in research rely on proxies to make a decision based on their wishes and preferences. However, patients rarely discuss their preferences about research and proxies find it challenging to determine what their wishes would be. While the process of informed consent has traditionally been the focus of research to improve consent decisions, the more conceptually complex area of what constitutes ‘good’ proxy decision-making for research has remained unexplored. Interventions are needed to improve and support proxy decision-making for research but are hampered by a lack of understanding about what constitutes decision quality in this context. A global increase in conditions associated with cognitive impairment such as dementia has led to an urgent need for more research into these conditions. The COVID-19 pandemic and subsequent necessity to conduct research with large numbers of critically ill patients has made this need even more pressing. Much of the empirical research centres on the desire to improve decision accuracy, despite growing evidence that authenticity is more reflective of the aim of proxy decisions and concerns about the methodological flaws in authenticity-focused studies. Such studies also fail to take account of the impact of decision-making on proxies, or the considerable body of research on improving the quality of healthcare decisions. This paper reports a concept synthesis of the literature that was conducted to develop the first conceptualisation of ‘good’ proxy decisions about research participation. Elements of decision quality were identified across three stages of decision-making: proxy preparedness for decision-making which includes knowledge and understanding, and values clarification and preference elicitation; the role of uncertainty, decisional conflict, satisfaction and regret in the decision-making process; and preference linked outcomes and their effect. This conceptualisation provides an essential first step towards the future development of interventions to enhance the quality of proxy decision-making and ensure proxy decisions represent patients’ values and preferences.

## Background

Adults who lack the ability to make decisions, even when supported to do so, rely on proxy decision-makers to make a range of decisions on their behalf [1]. This includes decisions about taking part in research where there are legal arrangements for the involvement for a proxy to make decisions about participation on the person’s behalf [2, 3]. Proxy decision-making can be challenging and those making decisions assume the responsibility for those decisions [1], however proxy decisions about research are especially complex [4]. In addition to the requirement for the proxy to receive and understand information about the study, the legal frameworks require their decision to be based on what, in the proxy’s view, the person’s wishes and feelings would be about participating in the study [2]—their ‘presumed will’ [3]. This reflects the normative ethical standard of substituted judgement. However, empirical evidence suggests that it can be challenging for proxies to determine these preferences in practice, which can lead to them experiencing a decisional and emotional burden as a result [5]. With the rising prevalence of conditions such as dementia [6], and more recently the challenge of conducting research involving patients who are critically ill with COVID-19, there is a growing need for proxy decision-makers in order to effectively carry out research with these populations.

Concerns about the ‘accuracy’ of proxy decision-making, including that proxies may incorrectly predict patients' preferences in one third of cases, have led to claims that this inability to predict incapacitated patients' preferences undermines the justification for using proxy decision-makers [7]. ‘Accuracy’ is defined as the proxy correctly inferring the person’s preferences [8] which problematically relies on the concept of there being one true preference. Studies highlighting inaccuracy in proxy decision-making predominantly use hypothetical scenarios to explore patient-reported preferences measured against proxy-reported decisions [8]. This is despite the counterfactual and other methodological issues raised by the use of hypothetical scenarios and treating the patient’s prediction about what they would wish as the ‘gold standard’ against which the proxy’s decision is assessed [4]. More recently, studies have explored ‘real life’ proxy decision-making, including about research participation, which suggest that authenticity rather than accuracy should be viewed as the basis for proxy decision-making [9]. Here, authenticity is viewed as the moral ideal of being ‘true to oneself’, although this is understood as being socio-culturally constituted and developed in dialogue with others, and the creation of a cohesive narrative [10]. Although others reject the reliance on narrative cohesion and argue that authenticity may also take into account the present-day settled preferences of people with impaired capacity, not just their past decisions [11]. This recognition of the importance of authenticity as the aim of proxy decision-making suggests that rather than pursuing accuracy-enhancing interventions to improve proxy decisions, or abandoning the involvement of proxies altogether, drawing on concepts identified in the considerable body of research into improving and supporting decision-making may be a more useful focus.

A number of interventions to improve proxy decisions about healthcare choices have previously been developed [12]. There has also been a rise in decision aids for patients making informed consent decisions about research participation [13]. To realise their goal, patient decision aids focus on three aspects: explaining the choice problem; providing evidence-based information on the relevant options and the advantages and disadvantages associated with those choices; and clarifying the personal values associated with each choice, usually by means of a values clarification exercise [14]. Concerns have arisen over whether such interventions do support informed and value-consistent choices, with the need for greater thinking about the normative assumptions underpinning consent decisions [14].

The first intervention intended to support proxy decision-making about research participation is being developed that focuses on improving decision-making processes beyond merely increasing ‘informedness’ [15]. However, as with interventions to improve decisions about research participation for oneself, the challenges around identifying the indicators of a good proxy decision, and how to evaluate whether a good decision has been made, remain. The lack of a definition of a ‘good’ decision is a critical barrier to developing highly effective decision support interventions [16]. In order to be effective, these interventions need to be guided by a clear definition of a decision quality, and have valid and reliable measurement approaches consistent with that definition [16].

The aim of this paper is to discuss, for the first time, what constitutes quality proxy decision-making for research, and for whom. A concept synthesis approach was used to analyse the phenomenon of proxy decision-making though an iterative process of identifying relevant normative concepts in the literature and empirical research [17]. This novel conceptualisation of good proxy decision-making for research is a necessary next step in the development and evaluation of interventions to enhance and support decision-making for research involving adults who lack capacity to consent. This concept synthesis forms part of a wider project to establish the core outcomes for use when evaluating interventions to improve proxy decisions about trial participation [18].

## Methodological approach to conceptualising surrogate decision-making quality

As ‘good’ proxy decisions about research participation have not previously been described, this synthesis explored this concept through examining related concepts such as normative standards of proxy decision-making, the basis for interventions to improve informed consent decisions, and what constitutes good healthcare decisions. The concept synthesis in this paper follows an established approach previously used to explore the concept of dignity in care for older people [19] and vulnerability in emergency care settings [20]. Concept analysis can identify the existing theoretical strands that define a concept and ultimately to tie and re-tie the conceptual knots to form a stronger, more coherent ‘tapestry of theory’ [21]. This result can present a coherent landscape which yields greater understanding of what is known about the concept [21].

Literature was reviewed to explore the conceptual aspects of decision quality relating to proxy decision-making for research. Searches of databases including MEDLINE, CINAHL, EMBASE, PsychInfo, and Cochrane Library were conducted by the author during the first half of 2019 using subject headings including decision making and informed consent, with searches restricted to English language papers but with no date limitations. As recommended in a concept synthesis, these searches were non-systematic, multidisciplinary and iterative in nature to ensure a breadth of literature was included [22]. Selection of the literature was conceptually driven to achieve adequacy [21], rather than being numerically driven or intended to be exhaustive. As the aim of a concept synthesis is to present a cohesive and theoretically informed conceptualisation [21], searches and subsequent analyses were conducted by one researcher, with discussions about data interpretation with a wider advisory group of researchers and lay members to ensure cohesion. In common with concept syntheses, an ‘inter-rater reliability’ approach to assess validity was not used nor was quality appraisal conducted.

Literature was reviewed from the perspectives of healthcare decisions (including decision science and decision support), informed consent, and proxy decision-making to identify elements which captured aspects of both proxy decision-making (i.e. the process) and decision quality (i.e. the decision itself) domains. Concepts of interest considered applicable to the inquiry were identified and assessed according to established principle-based criteria used in concept syntheses such as being pragmatic and logical [21]. Data relating to the concept were then extracted and reviewed alongside empirical data from our previous systematic review of ethical issues in proxy consent [4] and from our recent qualitative study which further developed an understanding of the ethical concepts involved [9]. Content concerning proxy decision-making in the empirical studies and normative literature was extracted as codes (containing a label and descriptor of the key concept) and synthesised to generate a concept matrix [19]. Having two criteria for defining a good decision (i.e. decision process and decision outcome) adds to the complexity of determining whether or not a single decision is good. However, they do not operate in isolation and improving decision-making involves measurement of both decision process and outcome criteria therefore both were included in the conceptualisation [23]. It should be noted that decision outcomes in this context includes those that are proximal (i.e. whether the proxy agrees to the person participating in the study or not) and distal (i.e. the consequences of that participation or non-participation).

**Fig. 1:**
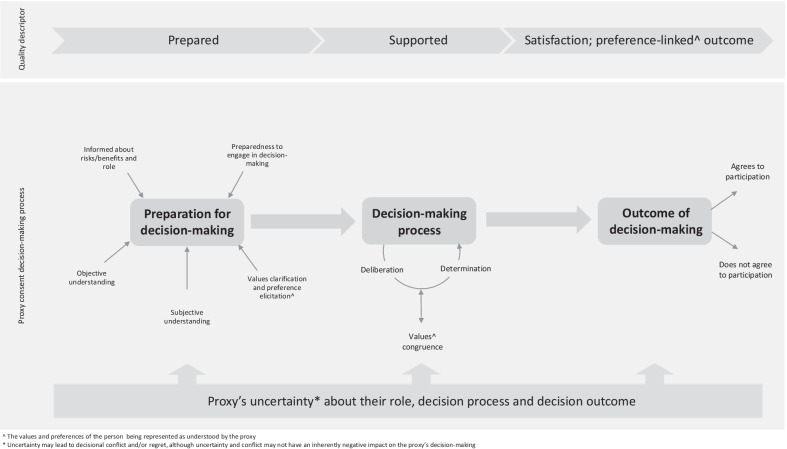
Proxy consent concept map

The elements identified that characterise decision quality are: proxy preparedness including knowledge, understanding, values clarification, and preference elicitation; the role of uncertainty, decisional conflict, satisfaction and regret in decision-making that seeks to achieve values-congruence; and preference linked outcomes and their effect. These elements are described in further detail in the sections that follow and are depicted in the concept map in Fig. 1 alongside the stages of proxy consent decision-making, with quality descriptors for each stage.

## Preparation for decision-making

Although not always recognised in the literature as a separate stage of decision-making, there is a pre-decisional process which occurs before engaging in the actual process of making a decision, where information is sought by the decision-maker, followed by appraisal of their knowledge sufficiency [24]. For proxy decision-making, this includes whether the proxy has sufficient knowledge about the values and preferences of the person they are making a decision on behalf of, their role as decision-maker, as well as material facts about the options being presented. Thus, as outlined below, in order for proxy decision-making to be viewed as being of good quality, the proxy should be adequately prepared to engage in decision-making and have sufficient understanding about their role, relevant information about the decision and the values and preferences of the person they are representing.

### Preparedness to engage in decision-making

In order to engage in making a good decision, the decision-maker must first recognise that a decision is needed (termed choice awareness), recognize the values‐sensitive nature of the decision, and understand that values affect the decision [25]. This is viewed as part of the preparation for decision-making [25] and includes preparation to communicate with a practitioner during a consultation and having awareness that their decision should be ‘informed’ [26].

### Proxy knowledge and understanding

Good quality decision-making is considered to require both objective and subjective understanding [27], such that the decision-maker *is* informed and *feels* informed. Those making proxy decisions about research should be informed about any potential risks and benefits associated with participation (and non-participation), as for informed consent given for oneself [2, 3]. However, unique to proxy decision-making is the requirement for the proxy to also be informed about their role and the basis for their decision—that they are representing the preferences and wishes of the person they care for and not on their own wishes and preferences. Our previous empirical research indicates that this important instruction is not always communicated through the written information provided to proxies [28] and so the normative ethical standard on which these decisions are made may not truly reflect a substituted judgement [9]. The literature is largely silent on the degree to which proxy decision-makers’ knowledge and understanding (both in terms of subjective and objective understanding) contributes towards a quality proxy decision, or how these elements could be either measured or improved. Decision interventions are often concerned with improving decision-makers’ confidence in their knowledge and their ability to make decisions or discuss options [25] and their role as proxy [29]. Confidence is seen as an important element within proxy decision-making and has been linked with improving accuracy of proxy decisions in previous empirical studies [4]. Whilst it is considered an important and modifiable factor in proxy decision-making, the exact nature of the relationship or causal mechanism between confidence and decision quality is less clear [29].

### Values clarification and preference elicitation

Good healthcare decisions require patients to consider both factual and probabilistic information, along with their personal values and preferences, and then choose the option that is most concordant with those values and preferences [16, 30]. Research participation decisions are also known to be preference-sensitive decisions, where good decisions are considered to have a match between the decision (participation or non-participation) and the features that matter most to the informed patient [31]. Whilst the terms values and preferences were not defined in the literature synthesised here, and indeed were often used interchangeably, values refers to high level constructs or attitudes such as truth-seeking or risk aversion, whereas preferences involves ranking the various attributes of options, such as drug side-effects which a patient prefers to avoid and hence will avoid that associated option [24]. Personal values are taken to be at least minimally consistent, stable and affirmed as a person’s own [32], whilst preferences are more fluid and malleable [33]. Preferences are constructed as individuals gain information and so are taken to be sensitive to different contexts and circumstances [24]. Consequently, values clarification is identifying the extent to which the benefits and risks of different options align with that person’s values, and preference elicitation involves identifying which options they would overall choose [34]. The aim of many decision aids is to clarify the patient’s values and determine their preferences towards the different choice options, in order to improve decision-making [35]. However, this is not a clear-cut task, in large part because they hinge on knowing two things that we cannot measure—a person’s ‘true’ values and what constitutes a good decision which is ultimately the aim of improving decision-making [35].

The assumption underlying the objective of values clarification and preference elicitation, that people have pre-existing, fully formed, stable preferences and values that merely need to be revealed through some elicitation process [35], has clear implications for proxy decision-making. As does the assumption that preferences known for previous particular decisions are a reliable and valid measure of the person’s long-term preferences, which may potentially lead to ‘projection bias’ [35], and that explicit, formal interventions to help articulate peoples’ values are superior to peoples’ implicit, intuitive approaches to knowing and identifying their preferences [35]. However, such assumptions are seen as problematic and lacking conceptual clarity [35], particularly regarding decisions about research where explicit pre-existing preferences are unlikely to have been expressed. One area of consensus is that clarifying values and preferences is neither a simple nor a clear-cut task [35].

Knowing the person’s values and preferences are important and relevant factors in proxy decision-making for research [4]. A number of empirical studies included in our previous systematic review explored whether proxies could accurately predict what the patient would decide about participating in a hypothetical research study [4]. Levels of ‘accuracy’ varied between studies, with some reporting high rates of discrepancy between enrolment decisions in patient–proxy dyads [4]. However, there are methodological flaws in these studies, including the use of hypothetical scenarios which limits the applicability of the findings to real-world proxy decision-making [4]. These studies conflate hypothetical and actual decisions and use the patient’s own ‘decision’ as the gold standard. In reality, the patient is making a prediction about their ‘decision’ and proxies are effectively being asked to match a guess [4]. The concept of accuracy ignores the counterfactual nature of proxy decision-making and assumes that preferences are fixed (rather than fluid and malleable) and that the proxy’s role is to uncover and present this ‘decision’. In contrast to aiming for decision accuracy, proxies who have experience of making decisions about research report that they aim to make a decision that is authentic to the values and preferences of the person they represent [9]. However, making authentic decisions isn’t always straightforward for proxies in practice, some of whom experience a decisional and emotional burden as a result [5].

In other studies included in the review, patients reported high levels of acceptability with family members acting as proxies to make decisions about research participation, although few had previously discussed research preferences [4]. Patients reported that *who* acted as their proxy was important, as well as *what* they decided [36–38]. Patients’ views about who they wished to represent them focused on the closeness of their relationship and how well the proxy knew them, and, in some cases, their previous experiences of the proxy making decisions for them [36–38]. There may also be common values and similar preferences between the patient and their proxy when they are close family members with shared formative experiences [5]. Proxies describe basing their decision-making primarily on their overall knowledge of the person’s values, wishes, past behaviours, and decisions, thus highlighting the importance of the proxy-patient relationship [39].

## Decision-making process

This stage in decision-making combines the processes of deliberating about decisions followed by the determination of a decision—described as the fulcrum of the event [24]. Deliberation involves using the information already obtained about the choices to imagine counterfactuals (*what if* scenarios), construct preferences, and forecast future affective responses (e.g. feelings of regret or disappointment) in readiness to make a decision [24]. Conceptions about what constitutes good quality informed consent decisions almost exclusively focus on knowledge and understanding without considering these other important aspects of decision-making [40]. As explored in this section, the difficulties experienced by proxies when attempting to make a decision, and the potential impact both for themselves and the person they represent, suggest that the quality of proxy decisions may be viewed through whether the proxy is supported to make a values-congruent decision. The importance of this congruence (also termed concordance) has its basis in autonomy which is considered to have two important elements—agency (the ability to make choices) [41], and authenticity (the ability to live a life of one’s own choosing) [42]. ‘Good’ decisions can be viewed as those where there is a close match between the chosen decision and the features that matter most to the informed patient [31]. The proxy aims to make a values-congruent decision during the deliberation stage of the decision-making process, and it is their uncertainty about whether participation in research is congruent with the person’s values that can lead to the decisional conflict they may experience.

### The role of uncertainty and decisional conflict

The decision-making literature in this synthesis draws heavily on the impact of decision-making on the decider. This includes uncertainty, which is defined as a subjective experience that can have either positive or negative effects [43] and which in decision-making can lead to feelings of conflict about the best course of action to take when making choices involving risk or uncertainty of outcomes [44]. Decisional conflict theory asserts that decision making is inherently a stressful event, surrounded by uncertainty and ambiguity leading to conflict as an undesirable state [45]. Decisional conflict can be seen as a measure of uncertainty during the deliberation stage of decision-making [40] and has long been the target of decision support interventions [25]. More recently, attention has turned to explore decisional conflict in proxy decision makers where there is a need to balance different concepts, such as preferences and interests, during decision-making [46]. The uncertainty experienced by proxy decision makers was illustrated in one study where proxies for patients in intensive care had higher scores on the uncertainty subscale of the Decisional Conflict Scale (DCS) than on all other subscales [47]. However, the assumption in decisional conflict theory that conflict is uniformly detrimental to decision making has been challenged by the view that it actually promotes appropriate deliberation and can have a positive impact on engagement in the decision-making process [35]. Under this view, rather than pathologising decisional conflict, it should be recognised as a normal response to uncertainty in complex decision-making contexts [35].

Proxy decision-making for research may be associated with significant emotional distress in settings such as critical care [48] at a time when many are experiencing anxiety and depression [49]. However, uncertainty is not high for all proxies [46] and while decisional uncertainty seems to enhance the complexity of proxy decision-making, it is not always correlated with expression of decision regret [50]. Regret is a complex negative emotion that we experience when we think that our current situation would be more favourable if we had chosen differently, in other words it is a counterfactual emotion arising through comparing current outcomes to what ‘might have’, ‘should have’, or ‘could have’ been [51]. It has been suggested that three conditions may enhance decisional conflict and decision regret in proxies for critical care patients: insufficient foreknowledge of the patient’s healthcare preferences; low decision-making self-efficacy associated with the probability of making a decision error, and a heightened sense of self-blame and disappointment related to the responsibility of being the patient’s proxy [50]. Decisional uncertainty may be particularly experienced in proxy decision-making for research as uncertainty is an inherent feature in research where the effectiveness of the treatment is unknown [52], and prior discussions about research preferences are relatively rare [5]. Generally, expressions of uncertainty appear to lead to non-action in terms of making a decision [53], which may be relevant to proxies being approached to make decisions in circumstances in which their uncertainty may affect their willingness to be involved.

The aim of interventions to improve decision-making are based on the conception of a ‘good’ decision having lower levels of decisional conflict and regret experienced by the decision-maker [54] and so increasing the likelihood of making an ‘effective’ decision [35]. Where ‘effective decisions’ are defined as those that are informed, consistent with personal values, and acted upon [44]. However, this relies on the assumption that uncertainty is undesirable and considered detrimental to decision-making [54]. A concern is that low decisional regret could result from perhaps a false view or hope, which is the very opposite of informed decision-making [54]. There are also anomalies in self-other differences in proxy decision-making involving risk [55] that suggest that rather than viewing proxy decision-making as a ‘decision under risk’ which requires known probabilities of outcomes, it should be viewed as ‘decision under uncertainty’ [56]. Under this alternative view, rather than treating decisional conflict as a state to be abolished, the message should be that living with uncertainty is normal and it is acceptable to feel ambivalent and uncertain about a potentially life-altering decision [35]. This suggests that rather than focusing on reducing decisional conflict, the focus should be on advancing a more realistic perspective on decision-making under uncertainty, that of managing or tolerating uncertainty [57] or perhaps a step further such as facilitating decision acceptance [35]. This may be achieved through interventions to support decision-making such as decision aids which are intended to reduce decisional conflict and the factors said to be contributing to uncertainty (e.g. feeling uncertain, uninformed, unclear about values, and unsupported in decision making) [25].

## Outcome of decision-making

The final stage of decision-making is that of having made the decision, together with any associated post-decisional outcomes [24]. In research participation decisions, the longer-term outcome of the decision may not be known immediately, for example whether the treatment received is effective or not or even whether they received an active intervention or placebo. As proxies can change their mind about participation at any point, unhappiness about a decision to participate, or with the outcome of that decision can lead to withdrawal from a study. Therefore, satisfaction with the decision is widely seen as a key part of determining whether informed consent decisions [58] and proxy decisions [8] are good. This will now be described further, alongside the dual aim that the decision made is aligned with the values and preferences of the person the proxy is representing.

### Satisfaction and regret in decision-making

Satisfaction with the decision experience can be viewed as comprising some (or all) of satisfaction with the preparation for decision-making, the process of decision-making, or the choice [25], and it may be aligned to the decision-maker’s desire to participate in that decision [53]. Satisfaction with a particular decision-making process (rather than the outcome alone) has been shown to predict patients’ levels of certainty that they will carry out the decision, demonstrating a link between satisfaction and action [53]. Satisfaction may also include more complex concepts such as whether the decision-maker is satisfied that the decision is consistent with their personal values, and satisfied that this was their decision to make [53]. In the literature around informed consent, participants’ lack of understanding about the nature of the clinical trial has been linked with lower satisfaction with their decision to participate, and may have ultimately led to regret about having joined [58]. The relationship between proxies being (or *feeling*) informed, being satisfied that a values-congruent decision has been made (albeit consistent with whose values) and experiencing decision regret as proxy decision-maker is unclear. Despite a lack of clarity around the mechanisms, measures of satisfaction, and value concordance are frequently used in the evaluation of proxy decision-making interventions [12].

One source of the complexity of decision-making is the multiplicity of regret types: process, option and outcome regret [59]. Joseph-Williams and colleagues have highlighted the importance of a distinction between the deliberation (process of arriving at a decision) and determination (referring to the actual decision and its consequence) stages of decision-making when determining whether a decision was ‘good’ or not [59]. Process regret involves feeling the decision process was poor, for example, not seeking information on all available options before making a decision, option regret simply involves regret about the choice made, and outcome regret involves a comparative evaluation and regretting that the outcome is poorer than the counterfactual outcome [59]. However, this relies on individuals understanding the counterfactual of what life would have been like had a different decision been made [54] which can be challenging. In such circumstances, counterfactual thinking extends to extrapolating from the patient’s prior preferences and values to determine what they would decide about participating if they were competent to do so.

In addition to decision regret, individuals may regret the role they played in the decision-making process, without actually regretting the choice made [60]. However, few studies have explored decision or role regret in proxy decision-makers, despite evidence that proxy decision-making differs from making a decision for oneself [61]. The ‘blame’ feature of regret may play a particular role in proxy decision-making where the impact of the decision is (largely) experienced by someone who isn’t responsible for making the decision. In a study of proxies for critically ill patients, decision regret affected more than two thirds (69%) of participants [50]. In another study, proxy decision-makers of critically ill patients reported low levels of decisional regret 1–2 months later, perhaps indicating that studies that evaluate proxy decision-making retrospectively could miss the decision-making burden that is more prevalent at the time the decision is made [47].

## Preference-linked outcomes

Preference linked health outcomes—that is whether a patient experienced the outcomes they preferred and avoided the outcomes they wanted to avoid—contrasts with values-choice congruence which is an aim of the decision-making process rather than necessarily related to outcome. Patients achieving the outcomes they desire is an important part of shared decision-making [62]. However, researchers have highlighted that a good decision cannot guarantee a good outcome [63], and that judging decision quality through taking outcomes into account can introduce *outcome bias* [64]. Retrospective information can also affect the judged probability of outcomes which, in turn, affects evaluation of the decision quality [64]. For example, a decision may be considered to be bad if we believe that bad outcomes were highly probable, and the decision was made regardless, referred to as *hindsight bias* [64]. This is particularly relevant to decisions about research participation where, by its very nature, there is uncertainty about the benefits and relative risks of the intervention being trialled.

The link between the outcomes of a proxy’s decision and the preferences of someone who lacks decisional capacity to consent, and who may not be aware of the outcome or its consequences, is less clear in terms of judging the quality of that decision. In studies exploring proxy decisions about treatment, knowing which treatment is consistent with the patient's preferences and that patients are treated consistent with their preferences and values was frequently cited as reducing the negative effect on proxies [61]. Preferences regarding outcomes in research are more complex as they require the proxy to have knowledge of the relative risks and benefits of participation and non-participation, which may invoke therapeutic misconception if any misunderstanding occurs [65], but also have knowledge about the person they represent and how *they* would value the various risks and benefits and outcomes. The range of potential benefits (including direct benefits to the person’s health as well as more altruism-based benefits) are viewed by proxies are being relevant to their decisions about research participation [5].Thus proxy decisions about research involve different or perhaps multiple forms of values-congruence and preference-linked outcomes, often in relation to achieving less tangible benefits such as altruism, than proxy decisions about treatment [61]. This complexity of multi-attribute decisions and the accompanying moral responsibility experienced by proxies may explain post-traumatic stress symptoms being found in 35% of family members of critically ill patients making decisions about research, compared with less than 10% in those involved in decisions about clinical care [66]. Therefore, whether any benefit is achieved (or not) as an outcome of the decision to participate would be part of the proxy’s decision experience, although uniquely in decision-making the effect of the outcome would not be experienced by the decision-maker themselves.

## Discussion

While conceptions of quality in healthcare decisions made by autonomous patients is a mature and well-explored area, research exploring proxy decision-making for healthcare is in its infancy and, although arguably more conceptually complex, proxy decision-making for research participation remains unexplored territory. This paper presents the first attempt to conceptualise a ‘good’ proxy decision about research through synthesising the normative literature and empirical evidence and identifying the elements that characterise a quality decision. Aligned with the three stages of decision-making, a ‘good’ proxy decision is one where the proxy is prepared and supported, feels satisfied and achieves a preference-linked outcome, and where they accept the uncertainty they may experience about their role, decision process and decision outcome.

At a conceptual level, the primary indicator of proxy decision quality should not be limited to the knowledge and understanding of the decision-maker. Rather, a broader context needs to be considered, including important dimensions of satisfaction, confidence, values-choice congruence, and preferences related to both the decision-making process and outcome of the decision. It is not clear whether a decision can be considered good if elements contained within it conflict and if so for whom. For example, where the proxy’s decision is not value-congruent (which would be considered to be a poor outcome) but the proxy themselves experiences high satisfaction and low regret (a good outcome) [16]. Little empirical guidance was found in the literature about how to evaluate the overall quality of proxy decisions, either in decisions about research participation or other decisions made on behalf of someone with impaired decision-making capacity. Establishing whether a decision is ‘good’ or not is further complicated by unresolved issues related to measurement [16] and definition. There is no consensus regarding the conceptualisation or operationalisation of related concepts such as satisfaction and regret, which leads to confusion about the distinction between them and makes it challenging to compare studies [58].

Beyond reviews of the literature, previous studies have primarily described the practice of proxy consent for research (such as [67]), or focused on the experience of making proxy decisions about research, including hypothetical decisions in dementia research [39, 68]. One study that conceptualised proxy decision-making for research in critical care settings reported decision-making as being multi-faceted and taking place in three sequential stages: (1) being approached; (2) reflecting on participation; and (3) making a decision [69]. Factors balanced by the proxy included how they were first approached and by whom, as well as balancing uncertainty about the study risks and benefits against the patients’ interests and wishes [69]. Whilst the sequential process reported in the study is reflective of the findings from this synthesis, the study was intended to be a descriptive characterisation of what proxy consent in critical care is, rather than identifying what constitutes quality decision-making as outlined in this paper. Similarly, studies that have sought to explicate the concepts that capture the quality of informed consent given for oneself have primarily focused on information provision and identifying knowledge-deficits [70], rather than considering other aspects of decision quality included here such as decisional conflict or regret [31].

Limitations of this concept synthesis include that it is based on assumptions in the normative and empirical literature that proxy decision-making is an individual endeavour, despite some evidence that proxy decision-making is relational and highly contextualised in practice [5, 68]. It also does not address the plurality of decision-making in practice, where decisions about research are not isolated from other decisions made on behalf of the person. The literature is also silent on how decision quality might be viewed from a broader health care provider or health care system perspective, where a similar plurality of decisions about research and treatment/care options will occur. Future work should seek to address these gaps in the literature and further develop the concepts and the relationships between them that has been proposed in this paper.

### Implications for future research

Further exploration is needed of proxies’ experiences of making consent decisions in contexts requiring multiple decisions, including proxies’ views about what constitutes decision quality in these contexts. This will also need to take account of the complexity of the legal frameworks underpinning proxy consent that differ between and within jurisdictions that has been described elsewhere [71, 72], and in differing cultural and religious contexts. Ethico-legal factors may include different legal bases for proxy decision-making, who can act as proxy, how the proxy perceives their role, as well as individuals’ and communities’ attitudes towards the acceptability of proxy consent.

Encouraging patients and their proxies to discuss preferences about future research participation in the event of impaired decision-making may increase proxy knowledge about relevant preferences [73] and thus improve values-choice congruence. This may be aligned with Advance Care Planning interventions in which patients are supported to make their wishes known about future care and treatment options [74]. There have been previous calls for research on the feasibility of developing a (non-binding) advance statement to enable people in the UK, such as those with dementia, to state their views and wishes regarding their future participation in research [75]. However, this will also need to be considered within the legislation surrounding advance decision-making that apply in different jurisdictions.

Further work is underway to develop a novel intervention to enhance proxy decision-making for research in the form of a decision support intervention [15]. Additional work is needed to establish how proxy decision quality can be measured in order to evaluate this and future interventions. The next steps are to undertake further work to gain consensus with relevant stakeholder groups, including patients and caregivers, on the most relevant and important outcomes (COnSiDER Study) [76]. This will then form a core outcome set against which interventions to enhance proxy decisions about research on behalf of adults who lack capacity to consent can be evaluated. Appropriate outcome measurement instruments will then need to be identified that capture key domains of proxy decision-making. Further normative exploration is needed to further develop this conceptualisation of proxy decision-making, and the implications for substituted judgement and related standards.

## Conclusions

Although only a first step, this work represents a move towards a greater understanding of ‘good’ proxy decision-making about research participation by adults unable to provide consent. This may lead to the development of interventions to enhance proxy decisions about research participation, and so address some of the challenges encountered by researchers seeking to conduct studies with patients with impairing conditions and those who are critically ill. However, this is a complex new and evolving area, and so further normative and empirical exploration will be needed.

## Data Availability

Not applicable as no data were collected for this paper.

## Abbreviation

DCS: Decisional Conflict Scale
